# The three‐axial gyroscope sensor detects the turning point between opening and closing phases of chewing

**DOI:** 10.1002/cre2.137

**Published:** 2018-11-30

**Authors:** Ichiro Minami, Alex Wirianski, Ryosuke Harakawa, Noriyuki Wakabayashi, Greg M. Murray

**Affiliations:** ^1^ Removable Partial Prosthodontics, Division of Oral Health Sciences, Graduate School Tokyo Medical and Dental University Japan; ^2^ Discipline of Physiotherapy, School of Allied Health, Faculty of Health Sciences Australian Catholic University New South Wales Australia; ^3^ Jaw Function and Orofacial Pain Research Unit, Faculty of Medicine and Health, Sydney Dental School University of Sydney New South Wales Australia

**Keywords:** jaw movement, jaw‐tracking system, mastication

## Abstract

Most devices measuring the kinematics of masticatory function are cumbersome to setup and not portable. Data collection would be facilitated, particularly in the elderly, if the device used for the objective measurement of mastication was easily transportable and simple to setup. Accelerometers and gyroscope sensors are lightweight and portable and may be useful alternatives. The definition of the turning point between the opening and closing phases of chewing is important for studies of associations between muscle activity and effects of perturbations. Measures of the mediolateral angle (specifically, the mandibular tilt from the lateral view) allow the detection of the turning point between the opening and closing phases. The aim was to determine whether a three‐axial gyroscope sensor can detect the turning point between opening and closing phases of chewing. Fourteen asymptomatic participants chewed gum while the output was recorded from a three‐axial gyroscope sensor (Seiko Epson, Japan) attached to the chin and a 6 degree‐of‐freedom electromagnetic jaw‐tracking device (Pollhemus, USA). Bland–Altman plots were used to assess the matching of the recordings made by the three‐axial gyroscope sensor and the jaw‐tracking device. The turning points between the opening and closing phases of chewing matched closely when recorded by a jaw‐tracking device and when using a three‐axial gyroscope sensor. A three‐axial gyroscope sensor can validly detect the turning point between the opening and closing phases during chewing of gum.

## 1 INTRODUCTION

Objective measurement of mastication is important not only for understanding normal masticatory behavior but also in the evaluation of functional capabilities. For example, in the elderly, a loss of chewing ability has been associated with sarcopenia (Murakami et al., 2014; Teixeira et al., 2014), a decline in activities of daily living (Kimura et al., 2013), and impaired cognitive functions (Teixeira et al., 2014). Most devices used for the objective measurement of mastication are cumbersome and time‐consuming to setup and require the participant to travel to the research unit or laboratory. Data collection would be facilitated, particularly in the elderly, if the device for measuring masticatory function was easily transportable and simple to setup. Recently, an accelerometer attached to the skin of the chin has captured jaw movement smoothness for the evaluation of movement variability (Minami et al., 2010; Minami et al., 2011; Minami et al., 2012; Yashiro, Yamauchi, Fujii, & Takada, 1999). This device is portable and easy to setup and use and with minimal interference to the participant. This system can detect the tooth contact phase of the chewing cycle, as well as jerk‐cost, an inverse measure of movement smoothness (Minami et al., 2010; Minami et al., 2011; Minami et al., 2012; Molenaar et al., 2012).

An important measurement to define is the turning point between the opening and closing phases of chewing. A number of studies have employed analyses of jaw movement or jaw muscle activity based on data where the chewing cycle has been divided into opening and closing phases (Minami et al., 2010; Minami et al., 2011; Molenaar et al., 2012; Sae‐Lee et al., 2008; Sae‐Lee, Wanigaratne, Whittle, Peck, & Murray, 2006; Yashiro et al., 1999; Zhao, Whittle, Murray, & Peck, 2012). These analyses have, for example, facilitated the characterization of the effects of noxious jaw muscle stimulation or food type on motor activity (Akhter et al., 2014; Peyron, Maskawi, Woda, Tanguay, & Lund, 1997; van der Bilt, 2011). However, the turning point between opening and closing phases has been technically difficult to define with accelerometers in general, and the requirement of an additional measure such as vertical jaw displacement has limited portability of the equipment required.

A three‐axial gyroscope sensor is commercially available and can be used to provide measures of jaw angles. This device may provide the turning point between opening and closing phases; however, this has not been tested. If a three‐axial gyroscope sensor can provide an output that can detect this turning point, then a portable system with both a three‐axial piezoelectric accelerometer and a three‐axial gyroscope sensor should be able to detect all of the main phases of the chewing cycle.

The aim of this study, therefore, was to determine whether a three‐axial gyroscope sensor can detect the turning point between opening and closing phases of chewing. The first hypothesis was that there is a close agreement between the turning point between the opening and closing phases as determined from the jaw trajectories and jaw angles that were derived from a jaw‐tracking device. The second hypothesis was that there is a close agreement between turning point between the opening and closing phases as determined from the trajectories of jaw movement and as determined from jaw angles derived from a three‐axial gyroscope sensor.

## 2 MATERIAL AND METHOD

This study was conducted in the Tokyo Medical and Dental University. Experimental procedures were performed at the dental chair, which were approved by the Tokyo Medical and Dental University Human Research Ethics Committee (No. 874).

### 2.1 A three‐axial gyroscope sensor

**Figure 1:**
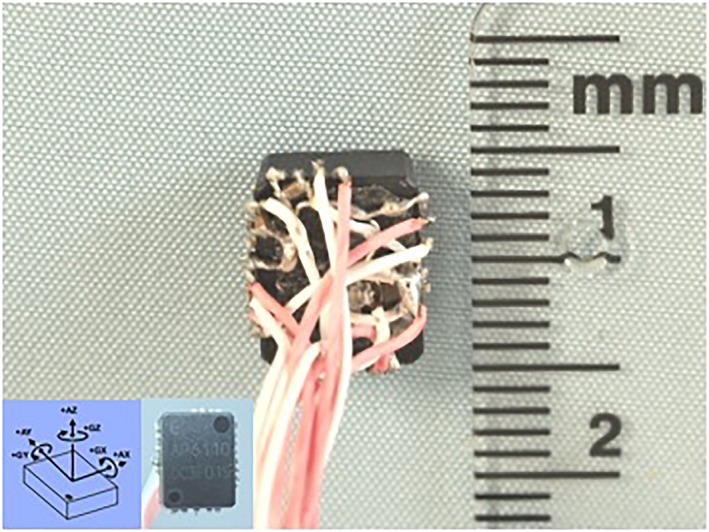
The 6 degree‐of‐freedom (6DOF) internal sensor (AP‐6110LR, Seiko Epson, Nagano, Japan). This sensor consists of a three‐axial accelerometer and a three‐axial gyroscope sensor. The circuit diagram is shown in the manual of this sensor. Ceramic chip capacitors and flexible cables (customized EPTFE cable, Junkosha, Tokyo, Japan) were soldered directly onto the sensor (custom made, Citizen Holdings Co., Ltd., Tokyo, Japan) according to the circuit diagram

The 6 degree‐of‐freedom (6DOF) internal sensor (AP‐6110LR, Seiko Epson, Nagano, Japan) was chosen for its small size (10.0 × 8.0 × 3.8 mm; Figure 1). This sensor consists of a three‐axial accelerometer and a three‐axial gyroscope sensor. Ceramic chip capacitors and extremely flexible cables (customized EPTFE cable, Junkosha, Tokyo, Japan) were soldered directly onto the sensor (custom made, Citizen Holdings Co., Ltd., Tokyo, Japan; Figure 1). The three‐axial gyroscope data were used in this study.

**Figure 2:**
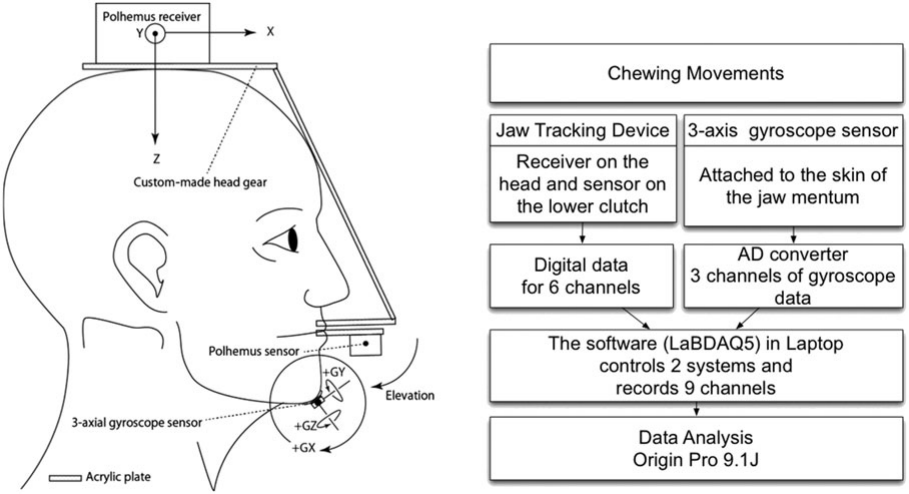
The method of attaching the jaw‐tracking device (Polhemus) and the three‐axial gyroscope sensor (AP‐6110LR) is shown in the left diagram, and an outline of the recording set‐up is shown in the right diagram. The Polhemus receiver was mounted parallel to the occlusal plane on the top of the head via an acrylic plate that was secured to the upper teeth. The Polhemus sensor was attached to an acrylic plate secured to the lower teeth via an acrylic clutch. The three‐axial gyroscope sensor was attached to the skin on the chin by means of a double‐sided tape. AD converter: analog to digital converter

The sensor was secured by means of double‐sided adhesive tape placed onto the surface of medical tape (Fixomull stretch; BSN Medical, Hamburg, Germany) that was attached to the skin on the chin of each participant, with the same method as described previously (Minami et al., 2010; Minami et al., 2012; Molenaar et al., 2012). The output from the three‐axial gyroscope provided angular velocity along three orthogonal axes (Figure 2). These three channels were fed through an analog to digital (A/D) converter (ADA16‐32/2(CB)F; Contec, Osaka, Japan) at a 1,200‐Hz sampling rate. The six data channels from the jaw‐tracking device (see below) were digitally fed into a personal computer and were stored on a hard disk with custom made software (customized LaBDAQ5‐CT Multi, Matsuyama, Ehime, Japan; Figure 2).

### 2.2 Jaw‐tracking device

A 6DOF electromagnetic jaw‐tracking device (Polhemus; 3Space Fastrak, Pollhemus Inc., Vermont, USA; Takanao, Kozawa, Yamashita, Igarashi, & Fujii, 2003) measured the position (X, Y, and Z Cartesian coordinates) and orientation (azimuth, elevation, and roll) of the jaw. It consists of a sensor and a receiver and provides a sampling rate of 120 Hz. It has a static accuracy of 0.03″ (0.08 cm) root mean square (RMS) for the X, Y, or Z receiver position, and 0.15° RMS for receiver orientation and resolution 0.0002″ of range (0.0005 cm of range) and 0.25° (Polhemus, 2012). Custom‐made headgear with an acrylic plate was secured on the buccal surfaces of the upper teeth, and the transmitter was secured to the top of the head and parallel to the occlusal plane. The sensor was secured to the lower teeth with a clutch made of acrylic resin and aligned parallel to the occlusal plane (Figure 2). The sensor was attached inverted in the X and Z direction in relation to the axes of the receiver (i.e., upside down and right side of the sensor was oriented to the left). Thus, angular changes of the sensor in the clockwise direction result in an increase in jaw angular elevation (Figure 2).

The position and orientation data (six channels) were digitally fed into a personal computer through a USB connection and were stored on a hard disk using custom software (customized LaBDAQ5‐CT Multi, Matsuyama, Ehime, Japan). This software employed linear interpolation that resulted in a 120 Hz sampling being converted to 1,200 Hz sampling in order for the Polhemus data to be measured simultaneously with the other sensors under the same sampling rate.

### 2.3 Participants

Informed, signed consent was obtained from 14 volunteers (six men and eight women; age range, 21–35 years; mean age ± *SD*, 27.2 ± 3.0 years).

The inclusion criterion was a complete permanent dentition except for the third molar teeth. Exclusion criteria were a history of temporomandibular disorders and symptomatic dental disease (e.g., periodontitis or dental caries).

### 2.4 Chewing tasks

Each participant was seated in an upright and relaxed position with his or her head unsupported and naturally oriented (Minami et al., 2010; Minami et al., 2011; Molenaar et al., 2012). Participants undertook five chewing sessions where each session consisted of one chewing sequence of a piece of softened gum (Xylitol Gum, Lotte Co., Ltd, Tokyo, Japan), measuring 13.0 mm width, 20.0 mm length, 6.1 mm depth, with the posterior teeth for 30 s. Before those experimental sessions were initiated, each participant was instructed to chew the gum until its viscosity became low and stable. In each experimental session, the participants were requested to chew on the gum bolus naturally (Minami et al., 2010; Minami et al., 2011; Molenaar et al., 2012). These five sessions were performed unilaterally on the preferred chewing side. For each participant, the five strokes after an initial three strokes were used for calculation, in accordance with previous studies (Minami et al., 2010; Minami et al., 2011; Minami et al., 2012; Molenaar et al., 2012), and the middle three sessions were used for further processing.

### 2.5 Data analysis

The stored data (three channels from three‐axial gyroscope sensor and six channels from the Polhemus) were analyzed with mathematical analysis software (Origin Pro 9.1J, Light Stone Co., Ltd., Tokyo, Japan). All the data from the Polhemus were converted so that the output represented incisal displacement against time together with angular data of the sensor attached to the lower teeth. The voltage data from the three‐axial gyroscope sensor were converted into units of angular velocity (i.e., GX, GY, and GZ in Figure 2), which was then integrated over time for each of the X‐ (mediolateral), Y‐ (anteroposterior), and Z‐ (superoinferior) direction series. The three‐dimensional orientation was then obtained.

Because most jaw movements are associated with rotations and translations of the temporomandibular joints, it is considered that the end point of the opening phase defined by vertical jaw displacement will be the end point of increasing elevation angle (arrow direction in Figure 2, mediolateral angle or X orientation). The turning point between the opening and closing phases of a chewing cycle was therefore defined by the software as the change in sign of vertical jaw displacement from the Polhemus (i.e., the position Z), elevation from the Polhemus (see Figure 2) and the X orientation (GX direction in Figure 2) from the three‐axial gyroscope sensor.

### 2.6 Statistical analysis

Each data set of the turning point between the opening and closing phases was analyzed with Bland–Altman plots, commonly used to appraise the agreement of between *n* measurements using a difference plot against the mean of the *n* measurements.

- A comparison of the turning points between the opening and closing phases as determined from the position Z of the Polhemus and as determined from elevation (see Figure 2) from the Polhemus.
- A comparison of the turning points between the opening and closing phases as determined from the position Z of the Polhemus and as determined from the X orientation (i.e., GX in Figure 2) from three‐axial gyroscope sensor.

## 3 RESULTS

**Figure 3:**
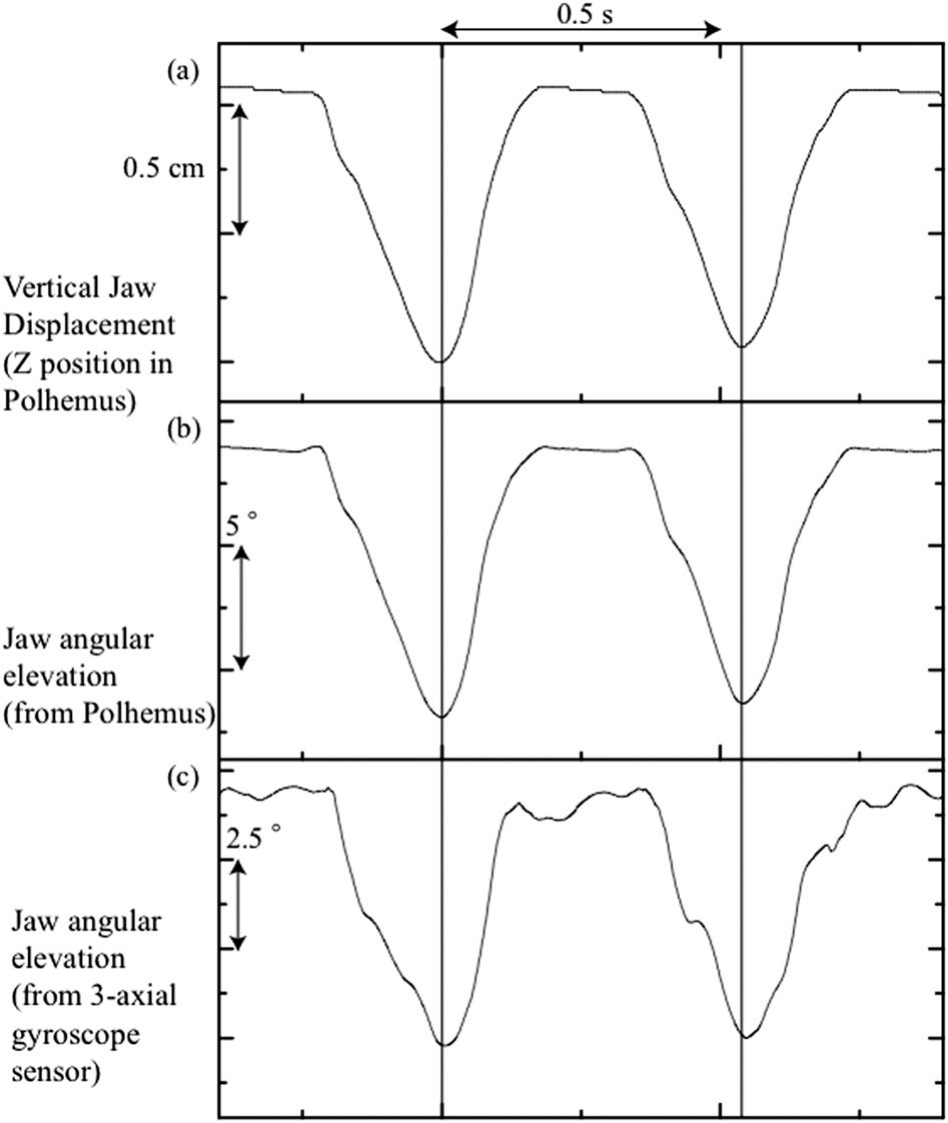
Typical data during two chewing cycles in a representative subject are shown as a function of time. (a) Vertical jaw displacement (Z position in Polhemus), (b) jaw angular elevation (from Polhemus), and (c) jaw angular elevation (from three‐axial gyroscope sensor). All y‐axes have been inverted in order to represent jaw opening in a downward direction. Vertical lines represent the turning point between the opening and closing phases

Typical data during two chewing cycles are shown in Figure 3. Vertical jaw displacement from the Polhemus (Figure 3a), elevation from the Polhemus (Figure 3b), and the X orientation from the 3‐axial gyroscope sensor (Figure 3c) are all shown as a function of time.

**Figure 4:**
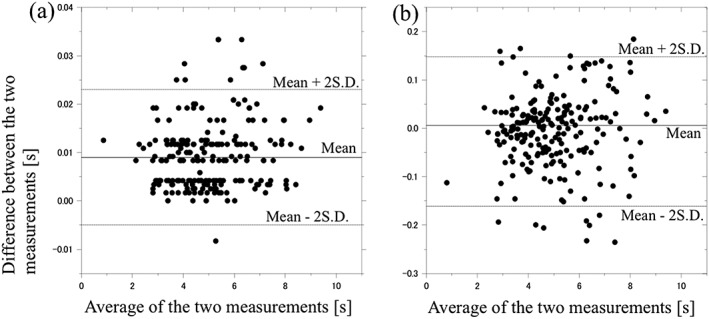
Bland–Altman plot figures of (a) differences between the turning points between the opening and closing phases of chewing as determined from position Z from the jaw‐tracking device (Polhemus) and as determined from the elevation from the jaw‐tracking device divided by the average of these two measures and (b) differences between the turning points between the opening and closing phases of chewing as determined from the position Z from the jaw‐tracking device and the X angular elevation from the three‐axial gyroscope sensor divided by the average of these two measures

Bland–Altman plots are shown in Figure 4. The left panel (a) shows the difference between time of occurrence of the position Z from the Polhemus and the elevation from the Polhemus divided by the average of these two measures. The right panel (b) shows the difference between time of the position Z from the Polhemus and the X orientation from the three‐axial gyroscope sensor divided by the average of these two measures. The mean difference and upper and lower limit agreement are shown in Table 1.

**Table 1:** Results of Bland–Altman plot for (a) the turning points(s) between the opening and closing phases of chewing as determined from the position Z of the Polhemus and as determined from elevation from the Polhemus and (b) the turning points(s) between the opening and closing phases of chewing as determined from the position Z from of the Polhemus and as determined from the X orientation (i.e., GX in Figure 2) from three‐axial gyroscope sensor

Statistic/Parameter	(a)	(b)
*N*	210	210
Mean ± *SD*	0.009 ± 0.007	−0.006 ± 0.077
Upper limit agreement (CI for upper limit)	0.023 (0.022 to 0.025)	0.149 (0.13 to 0.17)
Lower limit agreement (CI for lower limit)	−0.005 (−0.006 to –0.003)	−0.161 (−0.18 to –0.14)

*Note*. *N*: the total number of the strokes; *SD*: standard deviation; CI: confidence interval.

For the turning points between the opening and closing phases as determined from the position Z of the Polhemus and as determined from elevation from the Polhemus, the mean difference was 0.009 s. The 95% confidence interval for the lower limit of agreement was −0.003 to −0.006 s. For the upper limit of agreement, the 95% confidence interval was 0.022 to 0.025 s. Thus, the turning points between the opening and closing phases from the elevation angle of the jaw‐tracking device tend to give a larger reading than that from the position Z by between −0.005 and 0.023 s. The mean difference between these two measures was not significantly different from zero. There was no fixed or proportional (Figure 4) bias between these two measures. The 95% differences lay within the limits of agreement (mean difference ± 2 *SD*).

For the turning points between the opening and closing phases as determined from the position Z from of the Polhemus and as determined from the X orientation from three‐axial gyroscope sensor, the mean difference was −0.006 s. The 95% confidence interval for the lower limit of agreement was −0.18 to −0.14 s. For the upper limit of agreement, the 95% confidence interval was 0.13 to 0.17 s. The mean difference between these two measures was not significantly different from zero. There was no fixed or proportional (Figure 4) bias between these two measures. The 95% differences lay within the limits of agreement (mean difference ± 2 *SD*).

## 4 DISCUSSION

The first hypothesis was accepted, namely, that there is a close agreement between the occurrence of the turning points between the opening and closing phases of chewing as determined from the trajectories of jaw movement recorded by a jaw‐tracking device and as determined from jaw angles derived from the same jaw‐tracking device. Bland–Altman analysis revealed the agreement and the mean difference (0.008 s) between these two measurements was almost at the level of the sampling rate, namely, 120 Hz. The second hypothesis was also accepted, that is, there is a close agreement between the occurrence of the turning points between the opening and closing phases of chewing as determined from the trajectories of jaw movement recorded by a jaw‐tracking device and as determined from jaw angles derived from a three‐axial gyroscope sensor. The mean difference between the two measures was not significantly different from zero, and the differences lay within the range of limit of agreement (mean difference ± 2 *SD*). The data suggest that a three‐axial gyroscope sensor can validly detect the turning point between the opening and closing phases during chewing of gum.

The waveforms obtained from the three‐axial accelerometer (Minami et al., 2010; Minami et al., 2012) are also able to detect the boundaries between fast‐ and slow‐opening phases and fast‐ and slow‐closing phases (Ross et al., 2010), as well as the intercuspal phases. The present study demonstrates that the three‐axial gyroscope sensor is able to detect the turning point between the opening and closing phases of chewing. Therefore, two small sensors (i.e., accelerometer and gyroscope sensors) can be secured to the skin on the chin at the same time, and this set of two sensors should therefore be able to detect all turning points between all phases.

Jaw kinematic measuring methods that employ systems connecting the teeth to extra‐oral signaling devices can interfere with the subject's natural mastication. Further, the devices are usually expensive and complex to be used for clinical use and have been largely used only in laboratory settings. In this regard, this new method has many advantages given its portability and ease of use. These portable features help in evaluating functional capabilities such as in the elderly a loss of chewing ability associated with sarcopenia, a decline in activities of daily living, and impaired cognitive functions. As the sensor is secured to the skin, then movement of the skin in relation to the mandible could cause false data acquired by the sensor. However, it has been reported that both the mandible (in this case, the teeth) and the skin on the chin move in the same rhythm but with different magnitudes of displacement and velocity (Jemt & Hedegard, 1982). Further study is required to assess the kinematic measurement of jaw movement comparing the use of kinematic devices attached on the teeth to those secured to the skin overlying the chin.

Acceptance of the first hypothesis suggests that the measures of the mediolateral angle allow the detection of the turning point between opening and closing phases. Taking the feature of digital sampling into account, the data suggest that these two parameters are identical. Therefore, the healthy participants demonstrated that the elevation angle (see Figure 2) will increase in the opening phase during chewing, and it will decrease during the closing phase. This finding aids in the development of devices to divide the chewing cycle into phases because it is much more technically difficult for mobile devices to capture the position of the jaw than the angle of the jaw. Acceptance of the second hypothesis suggests that the three‐axial gyroscope sensor can validly detect the turning point between the opening and closing phases during chewing of gum. This has been technically difficult to detect with an accelerometer (Flavel, Nordstrom, & Miles, 2002; Minami et al., 2012). However, simultaneous measurement with another portable sensor such as an accelerometer and/or the use of EMG recording is recommended in order to confirm that the chewing cycle is being correctly captured.

In conclusion, these two sensors, secured by means of adhesive tape to the skin on the chin, establish a portable system that is able to divide the chewing cycle into the main phases of chewing. The accelerometer is able to detect the intercuspal phase, and the gyroscope sensor can detect the opening and closing phases. Future studies will focus on jaw movement smoothness and muscle activity in each phase of the chewing cycle for participants with pain or in older people and where there has been technical difficulty in collecting data given the lack of portability of jaw recording equipment.
